# Distal gastrectomy for gastric carcinoma in patients with diabetes mellitus: impact of reconstruction type on glucose tolerance

**DOI:** 10.20407/fmj.2018-004

**Published:** 2018-12-06

**Authors:** Kenichi Nakamura, Koichi Suda, Atsushi Suzuki, Masaya Nakauchi, Susumu Shibasaki, Kenji Kikuchi, Tetsuya Nakamura, Shinichi Kadoya, Kazuki Inaba, Ichiro Uyama

**Affiliations:** 1 Division of Upper GI, Department of Surgery, School of Medicine, Fujita Health University, Toyoake, Aichi, Japan; 2 Cancer Center/Department of Surgery, School of Medicine, Keio University, Shinjyuku, Tokyo, Japan; 3 Department of Endocrinology and Metabolism, School of Medicine, Fujita Health University, Toyoake, Aichi, Japan

**Keywords:** Stomach neoplasms, Reconstructive surgical procedures, Diabetes mellitus

## Abstract

**Objectives:**

Current evidence regarding metabolic surgery suggests that different types of digestive tract reconstruction can result in differences in postoperative glucose tolerance. This study evaluated the impact of Billroth I (B-I), Billroth II (B-II), and Roux-en-Y (R-Y) procedures on peri-operative glucose tolerance in patients with gastric carcinoma who had diabetes mellitus.

**Methods:**

A single-institution, retrospective cohort study was conducted using data from patients who underwent totally laparoscopic distal gastrectomy. These patients were grouped according to the type of reconstruction (B-I, B-II, or R-Y). After the operation, we addressed the changes in glucose tolerance—including changes in HbA1c levels, remission of diabetes, and overall effects of the treatment.

**Results:**

We studied 57 patients (B-I, n=32; B-II, n=17; R-Y, n=8). B-II and R-Y reconstruction improved HbA1c levels more than B-I. Notably, R-Y improved tolerance the most (B-I vs. B-II, *p*<0.001; B-I vs. R-Y, *p*<0.001; B-II vs. R-Y, *p*<0.001). The type of reconstruction (B-II and R-Y vs. B-I) and a pre-operative HbA1c ≥7% were the two significant independent contributing factors determining postoperative improvement in HbA1c, with odds ratio (OR) 8.437, 95% confidence interval (CI) 1.635–43.527, *p*=0.011; OR 16.5, 95% CI 3.361–81.011, *p*=0.001, respectively.

**Conclusions:**

Either R-Y or B-II should be considered the primary option for patients with gastric carcinoma and diabetes when glycemic control is insufficient.

## Introduction

Bariatric surgery has become very popular, with an increasing number of reports showing that it can induce weight reduction and also eliminate the symptoms of diabetes mellitus.^1–3^ Current evidence for metabolic surgery suggests that several types of digestive tract reconstruction, which can change the food pathway, may affect postoperative glucose tolerance.^4^ To date, among various bariatric and metabolic surgical procedures, sleeve gastrectomy and gastric bypass are the most common. In the sleeve gastrectomy approach, vertical transection of the stomach is performed, guided by an orogastric tube (size 36 Fr) placed along the lesser curvature for calibration. In the gastric bypass alternative, the stomach is transected in the upper portion. Gastro- and jejunojejunostomy are then created with the alimentary and biliopancreatic limbs.^4^

We often find that glucose tolerance of the patient who has gastric carcinoma and diabetes mellitus improves after gastrectomy.^5–7^ After distal gastrectomy, the alimentary tract is usually reconstructed using Billroth I (B-I, gastroduodenostomy), Billroth II (B-II, gastrojejunostomy), or Roux-en-Y (R-Y, separated alimentary and biliopancreatic limbs using gastrojejunostomy and jejunojejunostomy) procedures. Food flows from the remnant stomach to the duodenum in B-I. However, in B-II and R-Y, it passes from the stomach directly to the jejunum. The relationship between sleeve gastrectomy and gastric bypass is similar to that between B-I and B-II or R-Y in terms of the food pathway because gastric bypass and B-II/R-Y approaches exclude food from the duodenum and proximal jejunum. We hypothesized that different types of digestive tract reconstruction after distal gastrectomy for gastric carcinoma result in differences in postoperative glucose tolerance. To test this idea, we assessed the association between peri-operative changes in glucose tolerance in patients with gastric carcinoma and diabetes relative to the type of reconstruction employed.

## Methods

### Patients

Our study was conducted at a single institution. We performed a retrospective review of our prospectively maintained database comprising consecutive patients with resectable gastric carcinoma who underwent curative distal gastrectomy between 2008 and 2014. We excluded patients who underwent total gastrectomy, proximal gastrectomy and pancreaticoduodenectomy. The study included those patients who underwent treatment for diabetes pre-operatively. Patients were completely involved in the decision-making process, and informed consent for surgery was obtained from all patients. This study was approved by the Institutional Review Board of Fujita Health University.

### HbA1c levels

**Figure 1:**
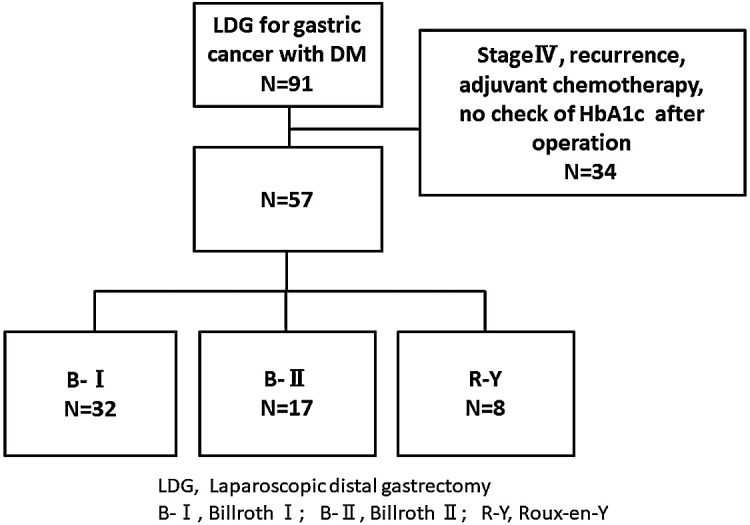
Flow diagram of patient enrollment

Peri-operative HbA1c levels were measured in the outpatient clinic at the following four time points (TPs): TP0, within 1 month before the operation; TP1, 1–3 months after the operation; TP2, 6–12 months after the operation; and TP3, 24–36 months after the operation. HbA1c levels were measured by high-performance liquid chromatography using the ADAMS A1c HA-8180V analyzer (ARKRAY, Inc., Kyoto, Japan). Patients in the analysis were classified into three groups according to the type of reconstruction (B-I, B-II, and R-Y) as shown in Figure 1. We assessed the changes in glucose tolerance after the operation, including the HbA1c levels, decreases in HbA1c levels (TP0–TP1, TP0–TP2, and TP0–TP3), remission of diabetes, and treatment achievement. Remission of diabetes was defined as an HbA1c level <6.5% with no medication.^8^ Treatment achievement of diabetes was defined as HbA1c level <7%, which the Japan Diabetes Society (JDS) sets as a target value to prevent complications of diabetes.^9^ To match the starting points (TP0) of the treatment achievement curve of the three types of reconstructions, we used the adjusted treatment achievement, in which we substituted HbA1c levels at TP1, TP2, and TP3 with either –1, 0, or 1 using the following cut-off points: –1 if the pre-operative HbA1c was <7% and the postoperative HbA1c was ≥7%; 0 if both the pre- and postoperative HbA1c were <7%, or both the pre- and postoperative HbA1c were ≥7%; and 1 if the pre-operative HbA1c was ≥7% and the postoperative HbA1c was <7% (Table 1). The “adjusted treatment achievement ratio” was calculated by dividing the total score at each TP by the number of patients.

### Surgical data

We assessed surgical outcome, including total operation time, estimated blood loss, postoperative complications, length of postoperative hospital stay, and clinicopathological characteristics. Early postoperative complications were defined as clinically significant issues occurring within 30 days following surgery that required surgical, endoscopic, or radiologic intervention, corresponding to a Clavien–Dindo (C–D) classification grade of III or more.^10,11^ Late postoperative complications, occurring on or after postoperative day 31, were defined as clinically significant complications corresponding to C–D grade II or more that required transfusion; central venous nutrition; or medications other than antiemetics, analgesics, antipyretics, or diuretics. The reason for applying C–D grade III or more for early postoperative complications is that we often use prophylactic antibiotics for findings consistent with mild inflammation without inspection early after the operation, because patients are especially vulnerable to infection during this period. Types of postoperative complications were classified in accordance with the Japan Clinical Oncology Group Postoperative Complication Criteria according to the C–D Classification ver. 2.0.^12^ Total operation time was calculated from the start of the abdominal incision through the completion of wound closure. Blood loss was estimated by weighing suctioned blood and blood-soaked gauze. In addition, we examined peri-operative changes in nutritional status, including body weight, body mass index, albumin, total protein, hemoglobin, and HbA1c, before and 1 year after the operation.

Various earlier papers have reported on the details of assessment of physical function, operative procedures, peri-operative management, extent of gastric resection and lymph node dissection, postoperative chemotherapy, and oncologic follow-up.^13–17^ Nutrition counseling for patients after gastrectomy as well as those with diabetes in accordance to its severity was conducted prior to discharge.

### Reconstruction of the digestive tract in laparoscopic distal gastrectomy: selection algorithm for type of reconstruction and procedure

**Figure 2:**
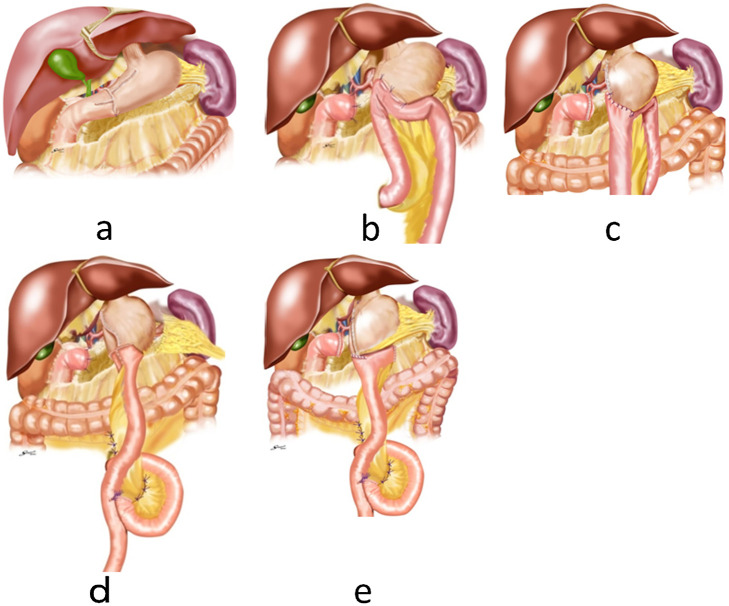
[a] Delta-shaped B-I anastomosis procedure.^21,22^ [b] The B-II procedure. The afferent loop was lifted to the lesser curvature of the remnant stomach and fixed by suture. Attention was paid to avoid a slack afferent loop to prevent the afferent loop syndrome without Braun’s anastomosis. [c] The B-II procedure in an isoperistaltic manner. [d] The antiperistaltic R-Y procedure. The jejunum was transected 25 cm away from the ligament of Treitz. After gastrojejunostomy was finished, jejunojejunostomy was created 30 cm anal from the gastrojejunostomy. [e] The isoperistaltic R-Y procedure. Regarding B-II and R-Y procedures, antiperistaltic anastomosis was used primarily, reserving isoperistaltic anastomosis for use when the remnant stomach would be too small after an antiperistaltic anastomosis to allow food passage straight through the abdominal esophagus, remnant stomach, gastrojejunostomy, and afferent jejunum.^18^ B-I, Billroth I; B-II, Billroth II; R-Y, Roux-en-Y.

B-I reconstruction was primarily selected as long as the remnant stomach reached the remnant duodenal bulb without excessive tension. When the remnant stomach was too small or the remnant duodenal bulb was too short, a B-II or R-Y procedure was chosen.^18^ B-II was also used because of comorbidity, age >80 years, and the operating surgeon’s preference. This was partly because B-II is simpler than the R-Y procedure, consuming less time to achieve anastomosis, and potentially leading to a lower incidence of leakage and other complications.^18,19^ R-Y was chosen because of pre-operative severe hiatal hernia, near-total gastrectomy preserving only fundus,^20^ and the operating surgeon’s preference. Reconstruction diagrams are shown in Figure 2. The delta-shaped B-I anastomosis was used for B-I.^21,22^ For the B-II and R-Y procedures, antiperistaltic anastomosis was used primarily, reserving isoperistaltic anastomosis for use when the remnant stomach would be too small after an antiperistaltic anastomosis to allow food passage straight through the abdominal esophagus, remnant stomach, gastrojejunostomy, and afferent jejunum. In the B-II procedure, the afferent loop was lifted to a lesser curvature of the remnant stomach and fixed by suture. Attention was paid to avoid a slack afferent loop to prevent the afferent loop syndrome without Braun’s anastomosis. In the R-Y procedure, the jejunum was transected 25 cm away from the ligament of Treitz. After gastrojejunostomy was accomplished, jejunojejunostomy was created 30 cm anal from the gastrojejunostomy.

### Factors associated with postoperative improvement in HbA1c levels

To determine the factors associated with postoperative improvement in HbA1c levels (TP0–TP3), univariate analyses were conducted using a wide range of variables, including age, sex, duration of diabetes mellitus, American Society of Anesthesiologists Physical Status (ASA-PS), use of neoadjuvant chemotherapy, pathologic Japanese Classification of Gastric Carcinoma (JCGC) stage, type of reconstruction (B-II and R-Y vs. B-I), hospital stay ≥13 days, short-term postoperative complications (C–D grade≥III), distant postoperative complications (C–D grade≥II), and total postoperative complications (C–D grade≥III)—as well as pre-operative body mass index, body weight, albumin, hemoglobin, oral antidiabetic agent use, insulin use, and pre-operative HbA1c ≥7%. Subsequent multivariate analysis was performed for the significant factors extracted in the univariate analysis.

### Statistical analysis

All analyses were conducted using IBM SPSS Statistics for Windows, Version 21.0 (IBM Corp., Armonk, NY, USA). Between-group comparisons were examined by a chi-squared (χ^2^), Mann–Whitney U- or Kruskal–Wallis test. One-way analysis of variance (ANOVA) was used to evaluate continuous variables among the three groups, and a χ^2^ or Fisher’s exact test was used to evaluate categorical variables. The comparison of peri-operative changes in HbA1c levels and the adjusted treatment achievement ratio of diabetes among the B-I, B-II, and R-Y groups were examined by repeated ANOVA tests. A univariate χ^2^ test and a multivariate logistic regression analysis with backward stepwise elimination were used to determine the factors associated with postoperative improvement of HbA1c. Considering the relatively small sample size, all variables with a significance level of *p*<0.05 in the univariate analysis for surgical outcomes were included as independent variables in the multivariate analysis. Data are expressed as medians (range) unless otherwise noted. A two-tailed *p* value of <0.05 was considered statistically significant. The Bonferroni correction was used to reduce the chances of obtaining false-positive results (type I errors) when multiple pairwise tests were performed on a single set of data.

## Results

### Patient background

Overall, records for 684 patients who underwent laparoscopic distal gastrectomy were reviewed; R0 resection was achieved in all patients, 91 of whom were pre-operatively diagnosed with diabetes by endocrinologists, according to the JDS criteria.^23^ Of these, 57 patients were enrolled in the study (B-I, n=32; B-II, n=17; R-Y, n=8). The remaining 34 patients were excluded from this analysis: 31 because they had factors that might affect glucose tolerance, including JCGC Stage IV disease, use of adjuvant chemotherapy, and/or disease recurrence, and three because their HbA1c was not measured after the operation. No cases of near-total gastrectomy, which may affect the postoperative glucose tolerance and the nutrition status, were included in this study. The total observation period was 67 (range 19–119) months.

Patient characteristics and demographic data are summarized in Table 2. In those with diabetes, there were no significant differences between the B-I, B-II, and R-Y groups regarding sex, age, BMI, or the duration of diabetes. However, there was a significant difference in the pathologic JCGC stage.

### Surgical outcome and short-term postoperative course

The surgical outcome and short-term postoperative course results are summarized in Table 3; there were no significant differences in the length of total operation time, estimated blood loss, length of postoperative hospital stay, or reoperation rate.

### Postoperative complications

Table 4 summarizes the postoperative complications; there were no significant differences in total morbidity (C–D grade≥III), complications within 30 days following surgery with C–D grade≥III, and complications on or after postoperative day 31 with C–D grade≥II.

### Peri-operative nutritional status

Peri-operative changes in nutritional status are summarized in Table 5. No differences were observed in body weight, body mass index, and total protein across the groups; however, in contrast, we saw slight changes in albumin, hemoglobin, and HbA1c levels.

### Peri-operative changes in glucose tolerance

**Figure 3:**
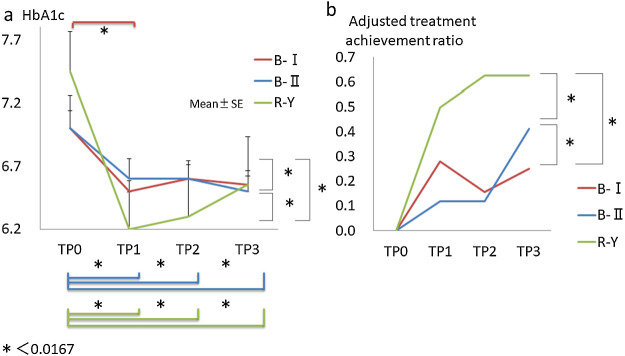
[a] HbA1c change (B-I vs. B-II vs. R-Y); and [b] adjusted treatment achievement ratio (B-I vs. B-II vs. R-Y). B-I, Billroth I; B-II, Billroth II; R-Y, Roux-en-Y; SE, standard error; TLDG, totally laparoscopic distal gastrectomy; TP0, within 1 month before surgery; TP1, from 1 to 3 months after surgery; TP2, from 6 to 12 months after surgery; TP3, from 24 to 36 months after surgery.

For 57 of the 91 patients, peri-operative HbA1c levels were measured in the outpatient clinic. Interestingly, we found that according to the within-group comparisons, HbA1c levels were significantly reduced postoperatively, irrespective of the type of reconstruction (B-I: TP0 vs. TP1, *p*<0.001; B-II: TP0 vs. TP1, *p*=0.003, TP0 vs. TP2, *p*=0.008, and TP0 vs. TP3, *p*<0.001; R-Y: TP0 vs. TP1, *p*<0.001, TP0 vs. TP2, *p*<0.001, and TP0 vs. TP3, *p*<0.001). Nonetheless, according to the between-group comparisons, improvement was observed in more patients in the B-II and R-Y groups than in the B-I group, with the greatest improvement seen in the R-Y group (B-I vs. B-II, *p*<0.001; B-I vs. R-Y, *p*<0.001; B-II vs. R-Y, *p*<0.001), as illustrated in Figure 3a. In terms of the reduction in HbA1c levels among the three types of reconstruction, there was a significant difference between the B-I and R-Y groups (TP0–TP1, *p*=0.006; TP0–TP2, *p*=0.001; TP0–TP3, *p*=0.033), but in contrast there was no change between the B-I and B-II groups (TP0–TP1, *p*=0.494; TP0–TP2, *p*=0.185; TP0–TP3, *p*=0.075) or between the B-II and R-Y groups (TP0–TP1, *p*=0.238; TP0–TP2, *p*=0.124; TP0–TP3, *p*=0.711). In our study, diabetes went into remission in 12 patients, and significant differences were observed between pre- and postoperative remission rates (pre-operation 3.5% vs. postoperation 21%, *p*=0.041). However, remission rates (TP0–TP3) did not vary postoperatively across the three groups (B-I vs. B-II, p=0.175; B-I vs. R-Y, *p*=0.070; B-II vs. R-Y, *p*=0.236), as detailed in Table 6. Remarkably, remission was achieved in all of the patients within 1 year postoperatively. The ratio of no medication use was altered from 22.8% (n=13) to 40.4% (n=23) (*p*<0.001). In eight patients who used insulin pre-operatively, only one in the B-I group withdrew from insulin treatment. The adjusted treatment achievement ratio was B-I: TP1, 28.1%, TP2, 15.6%, TP3, 25%; B-II: TP1, 11.8%, TP2, 11.8%, TP3, 41.2%; and R-Y: TP1, 50%, TP2, 62.5%, TP3, 62.5%. Patients who underwent B-II or R-Y had greater improvements in the adjusted treatment achievement ratio than those undergoing B-I, with patients undergoing R-Y showing the greatest improvement (B-I vs. B-II, *p*=0.005; B-I vs. R-Y, *p*<0.001; B-II vs. R-Y, *p*=0.005), as shown in Figure 3b.

### Factors associated with postoperative improvement in HbA1c levels

According to univariate analyses, we found that pre-operative HbA1c ≥7% (*p*<0.001), type of reconstruction (*p*=0.018), and hospital stay (*p*=0.04) was significantly associated with postoperative improvement in HbA1c (TP0–TP3) (Table 7). In addition, subsequent multivariate analysis showed three significant associations: pre-operative HbA1c ≥7%, type of reconstruction, and hospital stay. These data suggest that pre-operative HbA1c ≥7% and type of reconstruction were the independent contributing factors associated with postoperative improvement in HbA1c, with odds ratio (OR) 16.5, 95% confidence interval (CI) 3.361–81.011, *p*=0.001; OR 8.437, 95% CI 1.635–43.527, *p*=0.011, respectively (Table 7).

## Discussion

Our study clearly demonstrates that different types of digestive tract reconstruction after distal gastrectomy for gastric carcinoma in patients with diabetes results in differences in postoperative glucose tolerance in the long term. Although it has been reported that certain types of digestive tract reconstruction after distal gastrectomy in these patients can affect postoperative glucose tolerance,^5–7^ unfortunately few studies have shown long-term glycemic control. According to our multivariate analysis, the type of reconstruction and a pre-operative HbA1c ≥7% appear to be significant independent factors determining postoperative improvement in HbA1c. Although peri-operative changes in nutritional status were generally maintained within a clinically acceptable range irrespective of the type of reconstruction, glucose tolerance in the B-II and R-Y groups of patients with diabetes improved more than that in the B-I group. Notably, of the three types of reconstruction, R-Y improved the glucose tolerance to the greatest extent.

Recent studies on metabolic surgery have reported that gastric bypass improved the remission rate of diabetes mellitus more than sleeve gastrectomy,^4,24^ possibly because gastric bypass excludes food from the duodenum and proximal jejunum.^25^ This exclusion is believed to play an important role in reducing insulin resistance and improving diabetes control.^26^ There are two possible mechanisms at work here. First, the rapid delivery of nutrients to the lower intestine from the gastrointestinal bypass increases the stimulation of L-cells, which results in increased secretion of hormones that enhance insulin release and/or insulin action (for example, glucagon-like peptide-1), and a subsequent decrease in blood glucose levels. Second, gastrointestinal bypass reduces the secretion of upper gastrointestinal factors that decrease insulin secretion and/or promote insulin resistance. Reduction in the amount of these putative anti-insulin factors (or anti-incretins) increase insulin action, and therefore, improve the symptoms of diabetes mellitus.^27^ Therefore, R-Y or B-II reconstruction may be better for patients with diabetes whose glycemic control is insufficient because B-II/R-Y also excludes food from the duodenum and proximal jejunum. Moreover, it has been reported that having a longer distance between the gastrojejunostomy and the jejunojejunostomy improves glycemic control in patients with diabetes mellitus.^28^ Compared with B-II, the R-Y approach may be more suitable for such patients, especially those who use insulin, because jejunojejunostomy is not created in B-II. Further investigation on this point is warranted.

There are several of limitations to this study. First, it was conducted in a single institution using a nonrandomized design. The sample size was relatively small, and the observation period was relatively short. Therefore, the data may be biased, and overall results should be interpreted cautiously. Second, there was a between-group difference in patient characteristics, in part because of our selection algorithm for the type of reconstruction. Third, we used adjusted treatment achievement as one of the indicators of glycemic control in this study to match the starting points (TP0) of the treatment achievement curve of the three reconstruction alternatives. However, strictly speaking, apart from treatment achievement, the clinical significance of the adjusted treatment achievement has never been examined.

In conclusion, R-Y or B-II might be considered as the primary option for reconstruction when a patient with diabetes mellitus presents for distal gastrectomy for gastric carcinoma.

## Figures and Tables

**Table1:** Cut-off points and scores for adjusted treatment achievement

	Postoperative HbA1c <7%	Postoperative HbA1c ≥7%
Pre-operative HbA1c <7%	0	–1
Pre-operative HbA1c ≥7%	1	0

**Table2:** Characteristics and demographic data of patients

	Billroth I	Billroth II	Roux-en-Y	*p* value
No. of patients	32	17	8	
Sex, male:female	22:10	11:6	5:3	0.926
Age, years (range)	70 (53–86)	72 (55–84)	73 (57–80)	0.487
Body mass index, kg/m^2^ (range)	22.9 (18.7–29.5)	23 (15.4–32.1)	25.3 (21.1–32.7)	0.958
Pathologic JCGC stage (IA:IB:II:III)	27:3:1:1	7:4:5:1	7:0:0:1	0.011
Duration of diabetes, years (range)	10 (0–30)	4 (0–33)	7 (0.5–30)	0.897

JCGC, Japanese Classification of Gastric Carcinoma.

**Table3:** Short-term surgical outcome and postoperative course after distal gastrectomy for gastric cancer

	Billroth I	Billroth II	Roux-en-Y	*p* value
Short -term surgical outcome				
Total operative time, min (range)	303 (167–396)	337 (156–548)	269 (173–459)	0.436
Estimated blood loss, g (range)	32.5 (0–322)	32 (12–120)	27.5 (173–459)	0.741
Postoperative course				
Length of postoperative hospital stay, days (range)	14 (8–51)	13 (10–21)	11 (9–19)	0.110
Reoperation no.	0	0	0	

**Table4:** Postoperative complications of distal gastrectomy

	Billroth I	Billroth II	Roux-en-Y	*p* value
Total morbidity C–D grade≥III, n (%)	4 (12.5)	1 (5.9)	0	0.678
Within 30 days following surgery C–D grade≥III, n (%)	2 (6.3)	1 (5.9)	0	1.000
Anastomotic leakage	1 (3.1)	0	0	1.000
Pancreatic fistula	1 (3.1)	1 (5.9)	0	1.000
On or after postoperative day 31 C-D grade≥II, n (%)	2 (6.3)	1 (5.9)	0	1.000
Stenosis	1 (3.1)	0	0	1.000
Cholangitis	0	1 (5.9)	0	0.439
Adhesive small bowel obstruction	1 (3.1)	0	0	1.000

C–D, Clavien–Dindo classification.

**Table5:** Peri-operative changes in nutritional status

	Billroth I	Billroth II	Roux-en-Y	*p* value
Body weight pre-operation, kg (range)	60.3 (43.6–78.4)	61 (35.5–80)	65 (42–97.8)	
Body weight 1 year postoperation, kg (range)	55 (35.9–66.4)	55.5 (35.2–74)	64 (43.8–74)	0.314
Body Mass Index pre-operation, kg/m^2^ (range)	22.8 (18.7–29.5)	23 (15.4–32.1)	25.2 (21.1–32.7)	
Body Mass Index 1 year post operation, kg/m^2^ (range)	20.5 (15.3–25)	21 (15–25.3)	22.8 (21.1–27.8)	0.271
Albumin pre-operation, g/dl (range)	4.2 (2.5–4.6)	4.1 (3.1–4.5)	4.5 (4.1–4.7)	
Albumin 1 year postoperation, g/dl (range)	4.2 (2.8–4.8)	4.2 (3.3–4.9)	4.3 (0.2–4.36)	0.014
Total protein pre-operation, g/dl (range)	7.1 (5.2–8.3)	7 (6.3–8.5)	7.4 (7–7.9)	
Total protein 1 year postoperation, g/dl (range)	7.1 (5.7–8.5)	7.1 (6.1–7.9)	7.2 (6.2–7.6)	0.235
Hemoglobin pre-operation, g/dl (range)	12.9 (9.2–16)	11.7 (7.7–14)	14.9 (12.3–17.2)	
Hemoglobin 1 year postoperation, g/dl (range)	13.2 (9.2–15.3)	12.7 (8.7–14.8)	13.5 (10.2–14.6)	0.004
HbA1c pre-operation, % (range)	7 (5.5–9.3)	7 (6.2–9.5)	7.4 (6.8–9.5)	
HbA1c 1 year postoperation, % (range)	6.6 (5.7–8.4)	6.6 (5.8–7.9)	6.3 (4.9–7.9)	0.006

**Table6:** Pre- and postoperative use of antidiabetic medication

	Billroth I (n=32)	Billroth II (n=17)	Roux-en-Y (n=8)	*p* value
Pre-operation				
Insulin, n	6	2	0	0.531
Oral antidiabetic agents, n	18	10	7	0.267
Combined, n	0	1	0	0.439
No medication, n	8	4	1	0.646
Postoperation (TP3)				
Insulin, n	5	3	0	0.749
Oral antidiabetic agents, n	14	6	6	0.221
No medication, n	13	8	2	0.646

Remission (TP0–TP3), n	6	6	0	0.143
Remission for the first time at TP0, n	1	1	0	1.000
Remission for the first time at TP1, n	3	2	0	1.000
Remission for the first time at TP2, n	2	3	0	0.403
Remission for the first time at TP3, n	0	0	0	—

TP0, within 1 month before surgery; TP1, from 1 to 3 months after surgery; TP2, from 6 to 12 months after surgery; TP3, from 24 to 36 months after surgery.

**Table7:** Factors associated with postoperative improvement of HbA1c levels (TP0– TP3)

	Univariate analysis	Multivariate analysis	
	*p* value	*p* value	OR (95% CI)
Age	0.923		
Sex	0.76		
Pre-operative body mass index	0.12		
Pre-operative body weight	0.215		
Pre-operative albumin	0.674		
Pre-operative hemoglobin	0.773		
Pre-operative oral antidiabetic agents	0.422		
Pre-operative insulin	0.298		
Duration of diabetes mellitus	0.632		
ASA-PS	0.866		
Neoadjuvant chemotherapy use	0.702		
Pathologic JCGC stage	0.094		
Pre-operative HbA1c (≥7%)	<0.001	0.001	16.5 (3.361–81.011)
Type of reconstruction (B-II and R-Y vs. B-I)	0.018	0.011	8.437 (1.635–43.527)
Hospital stay (≥13 days)	0.04		
Short-term postoperative complications (C–D≥III)	0.662		
Distant postoperative complications (C–D≥II)	0.209		
Total postoperative complications (C–D≥III)	0.151		

ASA-PS, American Society of Anesthesiologists Physical Status; B-I, Billroth I; B-II, Billroth II; C–D, Clavien–Dindo classification; CI, confidence interval; JCGC, Japanese Classification of Gastric Carcinoma; OR, odds ratio; R-Y, Roux-en-Y; TP0, within 1 month before surgery; TP1, from 1 to 3 months after surgery; TP2, from 6 to 12 months after surgery; TP3, from 24 to 36 months after surgery.
